# A Boronic Acid-Based Glutamine Analog Forms a Covalent Adduct with Kidney-Type Glutaminase and Suppresses Triple-Negative Breast Cancer Cell Proliferation

**DOI:** 10.3390/biomedicines14051100

**Published:** 2026-05-13

**Authors:** Thiruselvam Viswanathan, Dinesh Devadoss, Achyuta Nagaraj, Barry P. Rosen, Hitendra S. Chand, Venkadesh Sarkarai Nadar

**Affiliations:** 1 Department of Cellular and Molecular Medicine, Herbert Wertheim College of Medicine, Florida International University, Miami, FL 33199, USA; tviswana@fiu.edu (T.V.); ddevados@fiu.edu (D.D.); brosen@fiu.edu (B.P.R.); hchand@fiu.edu (H.S.C.); 2 Center of Advanced Study in Crystallography and Biophysics, University of Madras, Chennai 600025, India

**Keywords:** triple-negative breast cancer, 2-amino-4-boronobutyric acid, glutaminase inhibitor, boronic compounds, non-proteinogenic amino acids

## Abstract

**Background**: Cancer cells exhibit metabolic reprogramming characterized by increased dependence on glutamine to sustain rapid proliferation and biosynthetic demands. Kidney-type glutaminase (KGA), which catalyzes the first and rate-limiting step of glutamine metabolism, represents a promising therapeutic target, particularly in triple-negative breast cancer (TNBC), an aggressive sub-type lacking effective targeted therapies. This study evaluated 2-amino-4-boronobutyric acid (ABBA), a boronic acid-containing glutamine analog, as a potential KGA inhibitor with anticancer activity. **Methods**: KGA inhibition was assessed using a fluorometric enzymatic assay. Cytotoxic effects were examined in multiple TNBC cell lines. Covalent docking and molecular simulation analysis were performed to characterize interactions between ABBA and the KGA active site. **Results**: ABBA potently inhibited KGA activity, with an IC_50_ of approximately 1.0 μM, demonstrating greater efficacy than several non-proteinogenic amino acid analogs. ABBA induced dose-dependent cytotoxicity across multiple TNBC cell lines, with pronounced sensitivity observed in basal sub-type cells and cellular sensitivity correlated with KGA expression levels. Expression of γ-glutamyl transpeptidase 1 (GGT1) was negligible, and, excluding any off-target effects, the observed anticancer effects are primarily attributed to KGA inhibition. Docking analysis indicated that ABBA forms a reversible covalent adduct with the catalytic Ser286 residue of KGA in a boronate tetrahedral geometry resembling transition-state mimics, while molecular simulation demonstrated stabilization of the complex through hydrogen bonding and electrostatic interactions. **Conclusions**: ABBA is a potent boron-based glutaminase inhibitor with therapeutic potential for targeting glutamine metabolism in TNBC. Further structural optimization and in vivo evaluation are warranted to advance ABBA toward therapeutic development.

## 1. Introduction

Cancer cells exhibit profound metabolic reprogramming relative to normal tissue, characterized by elevated aerobic glycolysis and dysregulated amino acid uptake and metabolism [1]. Among amino acids, glutamine plays a central role in supporting tumor growth and proliferation [2,3,4]. Rapidly proliferating tumor cells undergo metabolic reprogramming and increased reliance on glutamine as a carbon and nitrogen source for the tricarboxylic acid (TCA) cycle anaplerosis, nucleotide and lipid biosynthesis, redox homeostasis, and ATP production. Although glutamine can be synthesized de novo from glutamate, it becomes conditionally essential during rapid proliferation or under metabolic stress conditions, resulting in increased dependency in many malignancies [5].

Cancer cells increase glutamine uptake via transporters such as SLC1A5 (ASCT2), followed by mitochondrial conversion to glutamate by glutaminase (GLS), the first and rate-limiting step in glutamine catabolism [5,6,7]. Glutamate subsequently contributes to cellular metabolism via aminotransferases, which replenish amino acid pools, or through glutamate dehydrogenase, which generates α-ketoglutarate to sustain the TCA cycle and anabolic growth [7,8,9]. This metabolic reliance on glutamine has led to the classification of malignancies including glioblastoma, leukemias, lymphomas, lung cancer, pancreatic cancer, and triple-negative breast cancer (TNBC) as “glutamine-addicted” tumors [6].

In humans, glutaminase exists as two major isozymes: kidney-type glutaminase (KGA), encoded by GLS1, and liver-type glutaminase (LGA), encoded by GLS2, both of which exhibit distinct tissue distributions and regulatory mechanisms [10,11]. GLS1 produces two primary splice variants: the full length KGA and the shorter glutaminase C (GAC) [12]. These variants share an identical N-terminal sequence (1–550) but differ in their C-terminal regions (residues 551–669 for KGA and 551–598 for GAC) [12]. Elevated GLS1 expression has been reported in basal-like/TNBC and HER2 positive breast cancers and is associated with poorer disease-free survival, particularly in positive lymph node metastasis [13]. Luminal B tumors also exhibit elevated GLS expression compared to luminal A tumors and correlates with poor clinical outcomes [13].

Given the importance of GLS in cancer metabolism, several glutaminase inhibitors have been developed and classified according to their binding sites. Competitive inhibitors, such as 6-diazo-5-oxo-L-norlucine (DON), Acivicin, DRP-104, and JHU-083, target the catalytic site and broadly inhibit glutamine-utilizing enzymes [14]. Although DON and Acivicin demonstrated antitumor activity, their lack of selectivity and dose-limiting toxicity halted clinical development [14]. Prodrug approaches, including DRP-104 and JHU-083 [15], were designed to improve tumor selectivity and tolerance [16], alongside other DON prodrugs including Nedelcovych-13d [17], and Rais-5C [18]. In contrast, allosteric inhibitors such as BPTES bind to a hydrophobic regulatory site distinct from the catalytic center, inducing inactive conformations of GLS [19]. Improved derivatives, including CB-839 and IACS-6274, exhibit enhanced pharmacological properties, with IACS-6274 currently undergoing phase I clinical evaluation [14,20,21]. However, resistance to allosteric inhibitors, such as CB-839, has been reported in subsets of TNBC, limiting their clinical efficacy [22].

We previously demonstrated that hydroxyl arsinothricin (AST-OH) inhibits TNBC cell proliferation but is chemical instability due to oxidation of the trivalent arsenic center to the inactive pentavalent AST-OH [23]. Given the chemical similarities between arsenic and boron both are metalloids with analogous coordination properties. We explored boronic acid-based analogs as alternative glutaminase inhibitors. Boronic acids possess unique electronic and physicochemical properties including strong Lewis acidity and reversible covalent interactions with nucleophilic residues, making them attractive scaffolds in medicinal chemistry [24,25,26].

Here, we evaluate 2-amino-4-boronobutyric acid (ABBA), a glutamine analog boronic compound previously characterized as an inhibitor of gamma-glutamyl transpeptidase [27]. Using biochemical assays, cell-based studies, and covalent docking and molecular simulation analysis, we demonstrate that ABBA potently inhibits KGA activity and suppresses TNBC cell viability and forms a predicted covalent adduct with the catalytic Ser286 residue. These findings identify ABBA as a promising boron-based glutaminase inhibitor with potential therapeutic relevance in TNBC.

## 2. Materials and Methods

### 2.1. Chemicals

All chemicals and enzymes were purchased from Sigma-Aldrich Co. (St. Louis, MO, USA) unless otherwise specified. AST–OH was synthesized as previously described and used as a racemic mixture, with analyses focused on the L-enantiomer. Compound purity was confirmed by high-pressure liquid chromatography (series 2000, PerkinElmer, Waltham, MA, USA) coupled with inductively coupled plasma mass spectrometry (HPLC-ICP-MS, ELAN DRC-e; PerkinElmer). Trivalent R-AST–OH was obtained by chemical reduction in the pentavalent AST–OH. ABBA was obtained from Enamine US Inc. (Monmouth Junction, NJ, USA).

### 2.2. KGA Inhibition Assay

Glutaminase inhibition was assessed using a commercial GLS1 inhibitor screening kit (Cat# ab283389, Abcam, Waltham, MA, USA). The assay was conducted as described in the user manual. Briefly, in a 96-well black plate, all inhibitors at a final concentration of 10 µM were pre-incubated with KGA for 15 min. The reaction was initiated by the addition of substrate glutamine. The inhibition of all compounds was monitored by fluorescence at 535 nm excitation and 587 nm emission wavelengths using BioTek Synergy Neo 2 (BioTek instruments, Inc. Winooski, VT, USA) plate reader for up to 30 min. The initial rate was calculated for each sample, and the percentage of inhibition was determined using the no-inhibitor condition as 0% and 2 µM CB-839 as 100%. KGA was inhibited completely by CB-839 at 2 µm, and it was used as a negative control. The dose–response curves for ABBA and R-AST-OH (0.01–100 μM) were generated using Sigma Plot (version 10) and all experiments were performed in triplicate.

### 2.3. Cell Culture and Viability Assay

The TNBC cell lines HCC70 (ATCC^®^ CRL-2315TM), HCC38 (ATCC^®^ CRL-2314TM), HCC1937 (ATCC^®^ CRL-2336TM), and BT549 (ATCC^®^ HTB-122TM) were obtained from ATCC (Manassas, VA, USA) and cultured under recommended conditions. These cell lines were maintained in the recommended media under 5% CO_2_ in a humidified incubator at 37 °C. Cells were seeded at 3.0 × 10^4^ cells/well in 96-well plates and incubated for 24 h before being exposed to concentrations from 0.001 to 1000 µM of ABBA compounds for 72 h. Cell viability was then assessed using a CellTiter-Glo Luminescent Cell Viability Assay (Promega Corporation, Madison, WI, USA). The assay measured relative luminescence as an indicator of viability. The results showed dose-dependent cytotoxic effects of ABBA compounds on TNBC cell lines, with half-maximal inhibitory concentration (IC_50_) values calculated to assess potency.

### 2.4. Covalent Docking

The crystal structure of the DON molecule bound to KGA was derived for docking from the protein data bank (4O7D) [28]. Water molecules and the DON molecule were removed before docking, and only one monomer was used for analysis. Flare V10 software [29] facilitated protein and ligand preparation as well as docking evaluation. The Flare Protein Prep tool was utilized to prepare protein molecules for docking by removing water molecules and all heteroatoms not associated with the active site. Hydrogen atoms were added, and the protonation states of amino acid residues were assigned at physiological pH (7.0). The hydroxyl group of the active site residue Ser286 was selected as the center of the grid box with dimensions of 5.5 Å in each direction. The SMILES of the ABBA compound were obtained from PubChem, and its three-dimensional structure was generated in Flare. In the docking process, protein residues were static while the ligand was flexible. The docking grid, with 6 Å dimensions, was centered around the oxygen atom of Ser286, where DON binds. The top-ranked conformations were selected for further analysis, with a binding energy of −6.513 kcal/mol for ABBA and KGA. Docking results were visualized using PyMOL (Version 3.0) [30].

### 2.5. Molecular Simulation

The GROMACS (2022.5) [31] software suite was employed to perform the MD simulation. The protein and ligand were prepared using the CHARMM36-feb16 force field [32]. Ligand preparation involved the Avogadro molecular editor [33] and CHARMM General Force Field server (CGenFF 5.0) [34]. SER 286 was modified to its deprotonated form (SERD). An intermolecular bonded interaction between the side chain oxygen of SERD 286 and boron of ABBA was defined in the topology to represent the covalently bound protein–ligand complex. A rhombic dodecahedron box was defined, solvated with the CHARMM-modified TIP3P water model, and counterions were added to neutralize the complex. The prepared complex underwent steepest descent minimization, followed by equilibration in two ensembles: 1 ns under the canonical ensemble and 5 ns under the Isothermal–Isobaric ensemble, using V-rescale and Berendsen coupling. The system was constrained with LINCS and no dispersion correction. Electrostatic interactions were calculated using the particle mesh Ewald method, while a Verlet cutoff scheme with a force-switch modifier handled neighbor searching and Van der Waals interactions. The equilibrated system was subjected to a 100 ns production MD run with Parrinello-Rahman isotropic pressure coupling, saving energies and coordinates every 10 ps. Visualization and image generation were performed using UCSF CHIMERA [35] and VMD [36] visualization packages.

## 3. Results

### 3.1. Structural Analysis of Glutamine Analogs Identifies ABBA as a Potent KGA Inhibitor

**Figure 1:**
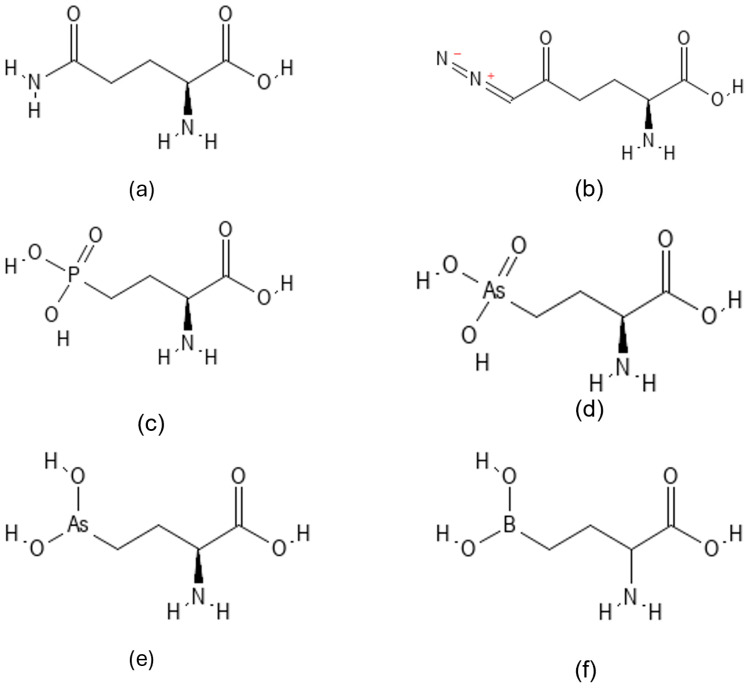
Chemical structures of glutamine and related glutamine analog inhibitors. (**a**) Glutamine; (**b**) 6-diazo-5-oxo-L-norleucine (DON); (**c**) 2-amino-4-phosphonobutyric acid (AP4); (**d**) pentavalent hydroxyarsinothricin (HAST); (**e**) reduced trivalent hydroxyarsinothricin (RHAST); and (**f**) 2-amino-4-boronobutanoic acid (ABBA).

Pharmacological inhibition of KGA (GLS) has emerged as a promising therapeutic strategy against cancer cells that utilize glutamine as an energy source for rapid growth and proliferation. The mitochondrial conversion of glutamine to glutamate by GLS represents the first and rate-limiting step in glutamine utilization, and specifically in TNBC, elevated GLS expression has been associated with cancer growth [37]. Figure 1 illustrates the chemical structures of glutamine (Figure 1a), and related glutamine analog inhibitors (Figure 1b–f). All compounds share a four-carbon backbone, and 6-diazo-5-oxo-L-norleucine (DON) (Figure 1b) serves as a well-characterized reference inhibitor. DON inhibits KGA and its isoforms by binding to the catalytic active site, and functions as a glutamine antagonist, thereby disrupting nucleotide and protein synthesis pathways that rely on glutamine [28]. Structural studies of glutaminase from *E. coli* and *B. subtilis* in complex with DON have identified the critical role of a conserved serine residue as the catalytic nucleophile [38] which corresponds to Ser286 in human KGA where the catalytic dyad composed of Ser286 and Lys289 is critical for enzyme catalysis [28]. However, despite its potency, DON lacks selectivity and inhibits multiple glutamine-dependent enzymes, including amidotransferases and glutamine synthetase, resulting in unacceptable toxicity in animal models that precluded further clinical development [14].

Previously, we demonstrated that the non-proteinogenic amino acids 2-amino-4-phosphonobutyric acid (AP4, Figure 1c) and hydroxyl arsinothricin (AST-OH) exhibit improved inhibitory activity against KGA relative to DON [23]. Notably, trivalent AST-OH displays potent anticancer activity in TNBC cell-based assays. However, the trivalent arsenic atom of AST-OH is chemically unstable and readily oxidizes to the pentavalent form (R=AST-OH, Figure 1d), which significantly diminishes its biological efficacy [23].

To overcome this limitation, we explored substitution of the arsenic atom in AST-OH with boron, a metalloid that shares similar coordination chemistry. Boronic acids possess a unique ability to function as strong Lewis acids due to the electron-deficient boron center, enabling reversible covalent interactions with nucleophilic residues [25]. Based on these considerations, we evaluated ABBA a structural analog of AST-OH in which the arsenic atom is replaced by boron. ABBA is known as a potent inhibitor of GGT1, and crystallographic studies have shown that L-ABBA binds covalently to the active-site and forms a covalent bond with threonine residue of GGT1 [27]. Given the shared catalytic serine nucleophile in KGA, we hypothesized that L-ABBA could similarly target the KGA active site and function as an effective glutaminase inhibitor with potential anticancer activity in TNBC.

### 3.2. ABBA Potently Inhibits KGA Activity

**Figure 2:**
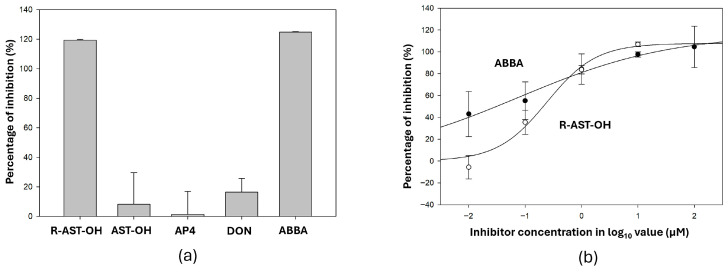
Inhibition of KGA activity by glutamine analogs. (**a**) Screening analysis of non-proteinogenic glutamine analog inhibitors at 10 µM using a fluorometric glutaminase inhibitor assay kit (Abcam). Inhibition percentages were calculated relative to the no-inhibitor control. (**b**) Dose–response curves for ABBA and R-AST-OH (0.01–100 µM). IC_50_ values were determined from nonlinear regression analysis. Data represent mean ± SD of triplicate experiments.

KGA inhibition by AP4, DON, AST-OH, R-AST-OH, and ABBA was evaluated using a fluorometric glutaminase assay. All compounds were initially tested at a final concentration of 10 μM, and the percentage of inhibition was calculated relative to the no-inhibitor control (Figure 2a). At 10 μM, both R-AST-OH and ABBA showed near-complete inhibition of KGA activity, whereas the AP4, DON, and AST-OH exhibited minimal inhibitory effects. To further quantify inhibitory potency, dose–response inhibition analyses were performed for R-AST-OH and ABBA across concentrations from 0.01 µM to 100 µM (Figure 2b). These experiments yielded IC50 values of 0.05 µM for R-AST-OH and 1.0 µM for ABBA. These results identify ABBA as a more potent KGA inhibitor than previously evaluated non-proteinogenic glutamine analogs.

### 3.3. ABBA Suppresses TNBC Cell Viability

**Figure 3:**
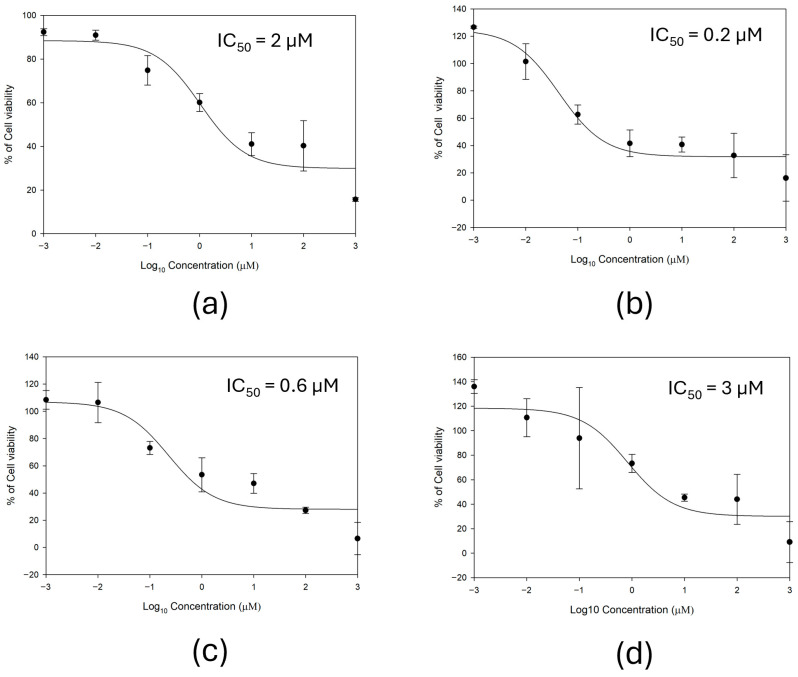
Effect of ABBA on TNBC cell viability. (**a**) HCC1937, (**b**) HCC70, (**c**) HCC38, and (**d**) BT549 cells were seeded in 96-well plates and treated with increasing concentrations of ABBA for 72 h. Cell viability was assessed using the CellTiter-Glo luminescent assay (Promega). IC_50_ values were calculated from dose–response curves. Data represent mean ± SD of triplicate experiments.

To evaluate antiproliferative effects of ABBA on cancer cells, we performed cell viability assays using a panel of TNBC cell lines representing distinct molecular sub-types: HCC70 (basal B), HCC38 (basal A), HCC1937 (basal A), and BT549 (mesenchymal). Cells were treated with increasing concentrations of ABBA for 72 h, and viability was assessed using the CellTiter-Glo luminescent assay (Promega), and IC_50_ values were calculated for each cell line (Figure 3). ABBA treatment resulted in a dose-dependent reduction in cell viability across all TNBC cell lines tested. At 10 µM, ABBA caused near-complete loss of viability in all four cell lines. Among these, HCC70 cells exhibited the greatest sensitivity to ABBA. The calculated IC50 values were 0.2 µM for HCC70, 0.6 µM for HCC38, 2 µM for HCC1937, and 3 µM for BT549 cells, indicating differential sensitivity among TNBC sub-types. Basal sub-type TNBC cells demonstrated greater sensitivity to ABBA compared to the mesenchymal sub-type.

**Figure 4:**
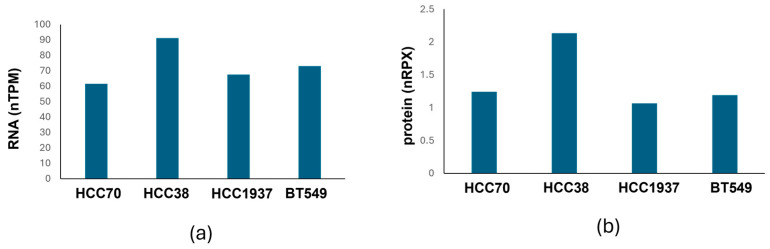
KGA (GLS1) expression levels in TNBC cell lines. (**a**) RNA expression levels (normalized transcripts per million, nTPM). (**b**) Protein expression levels (normalized protein expression, nRPTX). Data were obtained from the Human Protein Atlas.

To assess whether this variability was associated with KGA expression, protein expression data for KGA/GLS1 in the four TNBC cell lines were obtained from the Human Protein Atlas (Figure 4) [39]. Comparison of KGA expression levels with ABBA sensitivity revealed a trend toward increased sensitivity in cell lines with higher KGA expression. For example, HCC38 exhibited slightly higher KGA expression than HCC1937, consistent with their lower IC_50_ values. Although preliminary, these findings suggest that ABBA-mediated suppression of TNBC cell viability may be linked to KGA expression levels. However, additional studies across a broader panel of cell lines and mechanistic analyses are required to determine whether KGA expression is a predictive determinant of ABBA sensitivity and to elucidate the underlying molecular mechanisms.

### 3.4. Covalent Docking Supports Targeting of Catalytic Ser286

Boronic acid-containing compounds can form reversible covalent bonds with nucleophilic serine residues in proteins, acting as transition-state analogs through conversion from trigonal to tetrahedral geometry. DON has been shown to inhibit KGA by covalently modifying the catalytic Ser286 residue. During this process, the diazo group of DON is released, leaving 5-oxo-L-norleucine (ON) covalently bound to Ser286 and resulting in enzyme inhibition [28].

**Figure 5:**
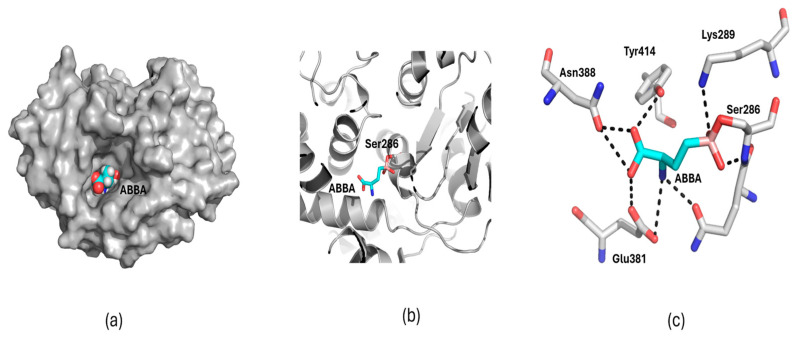
In silico covalent docking analysis of ABBA with KGA. (**a**) Surface representation of KGA showing ABBA bound within the active site. (**b**) Predicted covalent interaction between ABBA and the catalytic Ser286 residue. The boron atom adopts a tetrahedral geometry upon interaction with the Ser286 hydroxyl group. KGA is shown in cartoon representation. (**c**) Non-covalent interactions between ABBA and surrounding active-site residues. Hydrogen bonds are indicated by dashed lines. Oxygen, nitrogen and boron atoms are in red, blue and salmon color respectively. The carbon atoms in protein molecule is gray and in ABBA molecule is cyan.

Based on this established mechanism, we hypothesized that ABBA could similarly interact with the catalytic Ser286 residue via formation of a boron-serine adduct. To evaluate this possibility, covalent docking of ABBA to KGA was performed in silico using Flare software. Covalent docking simulations predicted stable binding of ABBA within the KGA active site, with a calculated binding energy of −6.513 kcal/mol (Figure 5a). The docking model indicated that the boron atom of ABBA forms a covalent bond with the hydroxyl side chain of Ser286, adopting a tetragonal geometry, consistent with boronic acid–serine interactions (Figure 5b).

In addition to the covalent bond, multiple non-covalent contacts contributed to the stabilization of the ABBA–KGA complex (Figure 5c). The amino group of ABBA forms hydrogen bonds with Gln285 and Glu381, while the hydroxyl group also engaged in hydrogen bonding with Glu381. The carbonyl group of ABBA established hydrogen bond interactions with Glu381, Asn388, and Tyr414. Furthermore, the positively charged Lys289 provided electrostatic stabilization of the negatively charged boronic species.

### 3.5. Molecular Simulation Illustrates the Stability of KGA–ABBA Complex

**Figure 6:**
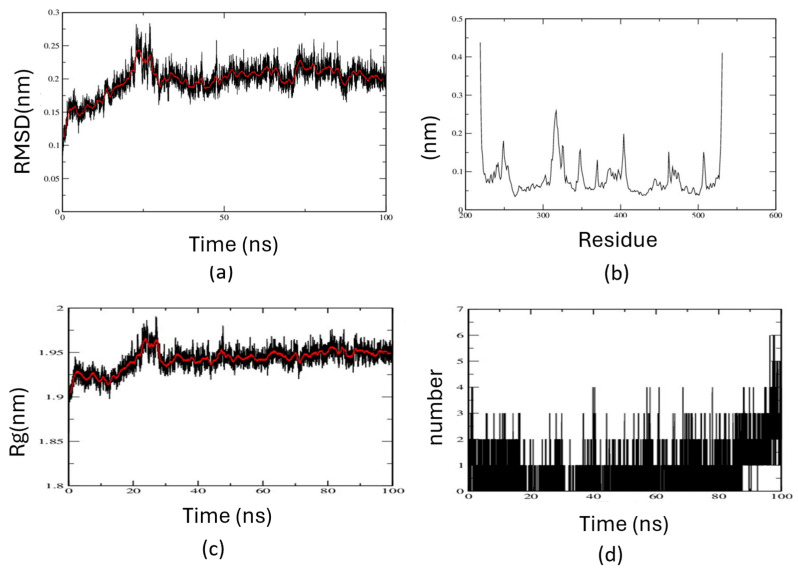
Molecular simulation of KGA–ABBA complex. (**a**) Root means square deviation of backbone of KGA (**b**) Root mean square fluctuation of residues of KGA. (**c**) Radius of gyration of KGA. (**d**) Number of hydrogen bonds formed over the course of 100 ns simulation. The running average is in red line.

The molecular simulation of the KGA–ABBA complex was conducted using the CHARMM36-feb16 force field and the GROMACS (2022.5) software suite. Structural stability and electrostatic interactions were analyzed through root-mean-square deviation (RMSD), root-mean-square fluctuation (RMSF), radius of gyration (RGYR), and hydrogen bonding between the protein and ABBA (Figure 6). The low RMSD value, settling around 0.2 nm after 25 ns, indicates minimal conformational changes. The RMSF plot identifies stable secondary structures and distinguishes loop regions and terminal residues. RGYR values around 1.95 nm confirm compactness throughout the simulation. Hydrogen bonding between ABBA and active site residues, though intermittent, may aid in aligning ABBA toward SER 286. Residues such as LYS289, TYR249, ARG387, ASN319, ASN335, GLN285, and others participate in hydrogen bonding with ABBA’s oxygens and nitrogen. The average total coulombic interaction energy for the simulation was calculated to be −516,819 ± 241 kJ mol^−1^. The total coulombic interaction energy was plotted along with its short-range, 1–4 and reciprocal components for the 100 ns simulation (Figure S1). The simulation movie is provided in the Supplementary Material. Collectively, these data support a binding mode in which ABBA engages the catalytic center of KGA through a combination of covalent and non-covalent interactions, consistent with its potent enzymatic inhibition. However, it requires experimental validation.

## 4. Discussion

Breast cancer remains a leading cause of cancer-related mortality among women worldwide. Clinically, breast tumors are classified based on the expression of the estrogen receptor (ER), progesterone receptor (PR), and human epidermal growth factor receptor 2 (EGFR2/Her2), which guide prognosis and therapeutic decision-making [40]. ER/PR-positive tumors are generally responsive to endocrine therapies, whereas Her2-positive cancers benefit from Her2-targeted treatments [41]. In contrast, the triple-negative breast cancer (TNBC), defined by the absence of ER, PR, and HER2 expression, represents the most aggressive sub-type and lacks effective targeted therapies [40]. TNBC accounts for approximately 10–20% of breast cancer cases, and is associated with increased metastatic potential, higher recurrence rates, and poorer clinical outcomes [42].

Cancer cells exhibit metabolic plasticity that enables adaptation to increased energetic and biosynthetic demands. Glutamine functions as a critical carbon and nitrogen source, supporting nucleotide synthesis, redox homeostasis, and anaplerotic flux into the tricarboxylic acid cycle. Accordingly, targeting glutamine metabolism has emerged as a promising therapeutic strategy, particularly for metabolically aggressive tumors such as TNBC. The KGA or GLS-1, which catalyzes the first and rate-limiting step in glutamine utilization, represents a key metabolic vulnerability in these cancers [6].

In this study, we demonstrate that the boron-containing non-proteinogenic amino acid ABBA inhibits KGA activity and suppresses TNBC cell viability. Compared to other glutamine analogs, ABBA exhibited enhanced inhibitory potency, consistent with its unique chemical properties. Boron, a metalloid distinct from carbon and silicon, exhibits strong Lewis acidity and readily forms reversible covalent interactions through trivalent coordination geometry. Upon interaction with nucleophilic residues, boron can transition from a trigonal planar to a tetrahedral sp^3^ configuration, enabling the formation of stable yet reversible covalent adducts.

This chemical behavior has been widely exploited in the development of boron-based enzyme inhibitors, particularly those targeting serine residues within catalytic active sites. Our in silico covalent docking analysis supports a mechanism in which ABBA forms tetrahedral boron–serine adducts with the catalytic Ser286 residue of KGA. Additional hydrogen bonding and electrostatic interactions further stabilize the ABBA–KGA complex, providing a structural basis for its potent inhibitory activity.

Functionally, ABBA induced marked cytotoxicity across multiple TNBC cell lines, with greater sensitivity observed in basal-like sub-types. Given that ABBA is a known inhibitor of GGT1, we assessed whether GGT1 inhibition contributed to the observed cellular effects. Analysis of transcriptomic and proteomic data demonstrated negligible GGT1 expression across all TNBC cell lines examined, indicating that the reduction in cell viability is unlikely to be mediated through GGT1 inhibition. These data suggest that ABBA’s anticancer activity may result from KGA inhibition alongside potential cytotoxic effects or off-target interactions, though these cannot be ruled out. Further studies, including metabolic profiling and direct assessment of GLS1 activity, are required to fully elucidate the mechanism of action. The docking analysis and molecular simulation work support the hypothesis of a covalent binding mode of ABBA with KGA, enabling it to inhibit the protein for an extended period, resulting in higher potency, prolonged duration of action, and potentially reduced or less frequent dosing. It is necessary to conduct further biochemical and structural studies to confirm this conclusion.

## 5. Conclusions

Together, our results identify ABBA as a promising boron-based glutaminase inhibitor that exploits glutamine dependency in TNBC. Further studies are warranted to evaluate in vivo efficacy, pharmacokinetic properties, selectivity, and potential therapeutic synergy with existing chemotherapeutic or metabolic-targeting strategies. This work further supports the development of boron-containing glutamine analogs as a novel class of metabolic inhibitors for the treatment of aggressive breast cancers.

## Data Availability

All materials are available from the corresponding author.

## Supplementary Materials

The following supporting information can be downloaded at: https://www.mdpi.com/article/10.3390/biomedicines14051100/s1. Figure S1: The total columbic interaction energy is plotted along with the reciprocal, 1–4 and short-range columbic interaction energies for the 100 ns simulation. Movie file: Molecular dynamics of KGA and ABBA complex.

## Abbreviations

The following abbreviations are used in this manuscript:

KGA	Kidney-type glutaminase
GLS1	Glutaminase 1
TNBC	Triple-negative breast cancer
ABBA	2-amino-4-boronobutyric acid
GGT1	γ-glutamyl transpeptidase 1
TCA	Tricarboxylic acid cycle
DON	6-diazo-5-oxo-L-norlucine
AST-OH	Pentavalent hydroxyarsinothricin
R-AST-OH	Trivalent hydroxyarsinothricin
AP4	2-amino-4-phosphonobutyric acid
