# Supplementary figures and images for: A Boronic Acid-Based Glutamine Analog Forms a Covalent Adduct with Kidney-Type Glutaminase and Suppresses Triple-Negative Breast Cancer Cell Proliferation
Source: Biomedicines. 2026 May 13;14(5):1100. doi: 10.3390/biomedicines14051100 (PMC13204110; doi:10.3390/biomedicines14051100)

## GROMACS Energies

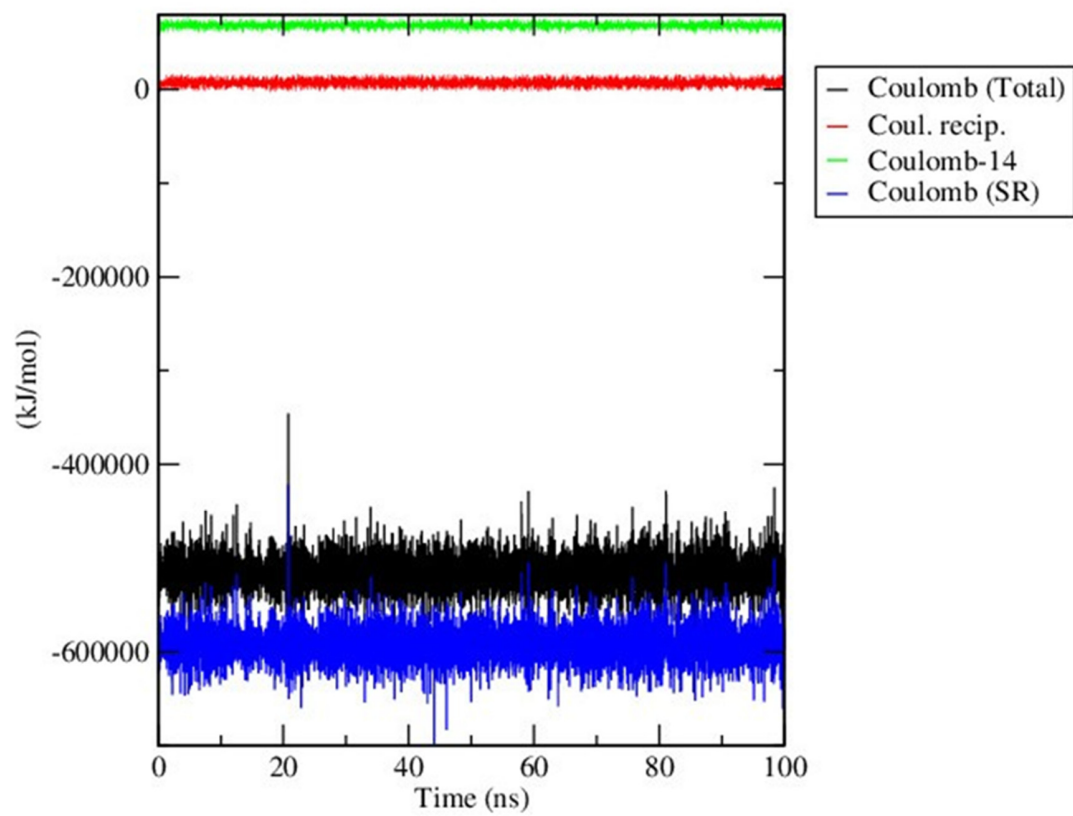

Supplement: Supplementary file 1 [file biomedicines-14-01100-s001.zip › Figure S1.pdf]
